# Supplementary material for: Three-dimensional X-ray diffraction imaging of dislocations in polycrystalline metals under tensile loading
Source: Nat Commun. 2018 Sep 17;9:3776. doi: 10.1038/s41467-018-06166-5 (PMC6141512; doi:10.1038/s41467-018-06166-5)
Supplement: Supplementary file 1 — Supplementary Information [file 41467_2018_6166_MOESM1_ESM.pdf]

Supplementary material for: Three-dimensional X-ray diffraction imaging of dislocations in polycrystalline metals under tensile loading

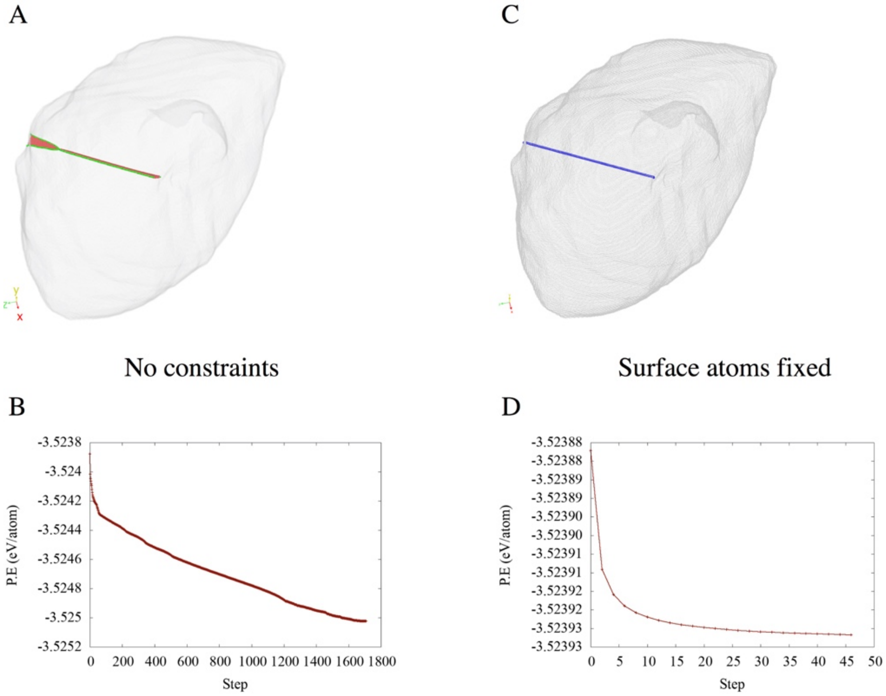

Supplementary Figure 1: Atomic structure and energy minimization. A Atomistic structure obtained following energy minimization with no constraints on atoms. C atomic structure obtained following energy minimization with surface atoms fixed. B,D show potential energy per atom as a function of minimization step. The dislocation structure was extracted using the DXA tool which is part of the OVITO visualization tool.[1,2]

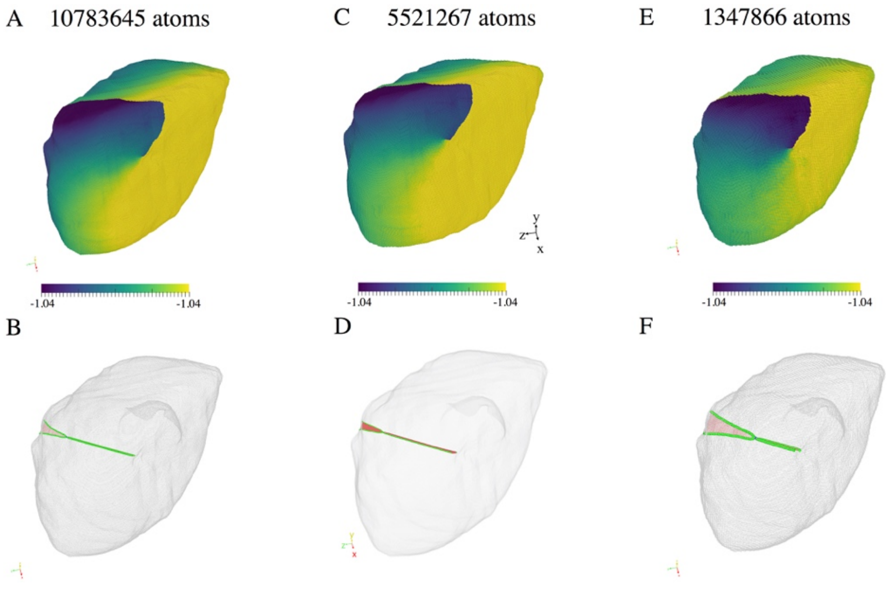

Supplementary Figure 2: Influence of size on simulation results. Displacement field and dislocation structure obtained following energy minimization for models scaled down by a factor of 4 (A,B), scaled down by a factor of 5 (C,D) and scaled down by a factor of 8 (E,F). In all cases, the observed

displacement fields and atomic structure of the dislocation are quite similar. The dislocation structure was extracted using the DXA tool which is part of the OVITO visualization tool. [1,2]

A Experimental data B Initial atomic structure C Relaxed atomic structure

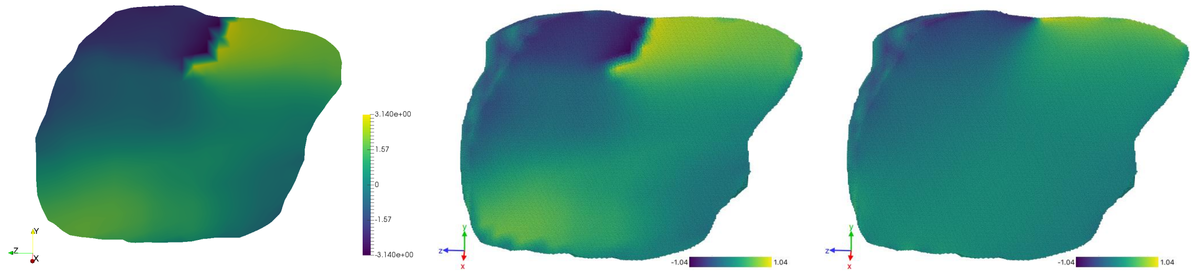

Supplementary Figure 3: Importance of neighbouring grains' constraints. A Experimentally measured structure and phases, coloring is by recovered phase in radians. B initial atomic structure (scaled by a factor of 5) where the atoms are displaced along the Q vector to match the experimentally observed displacement field. Color is by atomic displacement in Å. C atomic structure obtained following energy minimization, color is by atomic displacement in Å.

Supplementary References:

1. Stukowski, A., Bulatov, V. V & Arsenlis, A. Automated identification and indexing of dislocations in crystal interfaces. *Model. Simul. Mater. Sci. Eng.* **20,** 85007 (2012).
2. Stukowski, A. Visualization and analysis of atomistic simulation data with OVITO–the Open Visualization Tool. *Model. Simul. Mater. Sci. Eng.* **18,** 15012 (2010).
